# Retraction: Phenotypic characterization of exotic tomato germplasm: An excellent breeding resource

**DOI:** 10.1371/journal.pone.0274230

**Published:** 2022-09-14

**Authors:** The PLOS ONE Editors

The *PLOS ONE* Editors retract this article [1] because it was identified as one of a series of submissions for which we have concerns about authorship, competing interests, and peer review. We regret that the issues were not addressed prior to the article’s publication.

All authors did not agree with retraction.
